# Blood-Brain Barrier Disruption Induced Cognitive Impairment Is Associated With Increase of Inflammatory Cytokine

**DOI:** 10.3389/fnagi.2018.00129

**Published:** 2018-05-07

**Authors:** Jieli Geng, Liping Wang, Linyuan Zhang, Chuan Qin, Yaying Song, Yuanyuan Ma, Yajing Chen, Shengdi Chen, Yongting Wang, Zhijun Zhang, Guo-Yuan Yang

**Affiliations:** ^1^Department of Neurology, Shanghai Renji Hospital, School of Medicine, Shanghai Jiao Tong University, Shanghai, China; ^2^Department of Neurology, Shanghai Ruijin Hospital, School of Medicine, Shanghai Jiao Tong University, Shanghai, China; ^3^Neuroscience and Neuroengineering Research Center, Med-X Research Institute and School of Biomedical Engineering, Shanghai Jiao Tong University, Shanghai, China

**Keywords:** cognitive impairment, blood-brain barrier, diabetes, tight junction, Morris water maze

## Abstract

Patients with diabetes suffer the higher risk of dementia and the underlying pathological mechanism of cognitive dysfunction in diabetes is not fully understood. In this study, we explore whether the cognitive impairment in the diabetic rat is associated with increased blood brain barrier (BBB) permeability and the change of the inflammatory cytokine. Experimental diabetic rats were induced by single intraperitoneal injection of streptozotocin (STZ). Cognitive function was evaluated by Morris water maze in the normal and the diabetic rats, respectively. The spatial acquisition trials were conducted over five consecutive days and the probe test was performed on day 6, followed by working memory test on the next 4 days. Escape latency was recorded in the acquisition trials and working memory test; time spent in the target quadrant and the number of crossing the former platform were recorded in the probe test. BBB permeability was assessed by measuring the extravasation of IgG. The image of occludin and claudin-5 staining by a confocal microscope were acquired to measure the gap in the tight junction. Cytokines TNF-α, IL-1β and IL-6 mRNA expression were further examined by Real-time PCR. The time spent in the target quadrant within 30 s decreased in the 8-week STZ rats compared to that of the normal rats (*p* < 0.05), while no difference was seen in the performance of working memory between the diabetic and normal rats. IgG leakage significantly increased in the brain parenchyma of the 8-week STZ rats compared to the normal rats (*p* < 0.05). The immunostaining of occludin and claudin-5 suggested the gap in the tight junction increased in the 8-week STZ rats compared to the normal rats (*p* < 0.05). Moreover, TNF-α and IL-6 mRNA also increased in the brain of 8-week STZ rats compared to the normal rats (*p* < 0.05). These results suggested that loss of BBB integrity might contribute to progressive impairment of cognitive in the diabetic rats. The increase of TNF-α and IL-6 expression might trigger the disruption of BBB in the brain, which eventually caused cognitive impairment in the 8-week STZ rats.

## Introduction

The increasing incidence of diabetes mellitus has given rise to the public concern and related studies worldwide (Mathers and Loncar, 2006; Vos et al., 2012). Much attention has been given to its macrovascular complications such as cardiovascular and cerebrovascular disease. Recently several clinical studies demonstrated that patients with diabetes suffered the higher risk of “any dementia” than those without diabetes (Ott et al., 1999; Biessels et al., 2006; Pasquier et al., 2006), and this phenomena caused the extensive studies to explore the effect of diabetes on cognitive impairment. High glucose upregulated the expression of multiple inflammatory cytokines, including tumor necrosis factor-α (TNF-α), interleukin-1β (IL-1β) and interleukin-6 (IL-6), which might be linked to the pathogenesis of the complications of central nervous system in diabetes (Wang et al., 2012). Inflammatory response was also mediated the mechanism of BBB damage (Fernandez-Lopez et al., 2012; Uchida et al., 2017). However, the underlying pathogenesis of cognitive dysfunction in diabetes is not fully understood.

Cognition refers to the psychological process of understanding things. Cognitive function is composed of multiple cognitive domains including memory, calculation, spatial orientation, structure ability and executive ability, language comprehension and expression (Oatley, 1981; Dolan, 2002). The influence of diabetes on cognitive domains varied in clinical and experimental studies (Sadanand et al., 2016). A battery of screening tests for cognitive impairment in the clinical setting include memory, attention, language, and visuospatial or executive functioning, etc. The Morris water maze (MWM) test is commonly used to evaluate the cognitive function in rodents, which is reproducible and quantitatively countable (Vorhees and Williams, 2006; Webster et al., 2014).

Blood brain barrier (BBB) is constituted by the endothelial cells of cerebral microvessel, pericyte, end-foot of astrocyte and basal membrane in brain and spinal cord. The derangements of endothelial structure and function have been considered as an essential pathogenesis in diabetic microangiopathy. Microvascular disorder caused by diabetes could lead to the BBB impairment integrity and increased BBB permeability *in vitro* and *in vivo* (Chehade et al., 2002; Huber et al., 2006; Hawkins et al., 2007a). BBB permeability showed a progressive increase from 28 to 90 days after streptozotocin (STZ) injection in rats (Huber et al., 2006). It is noted that the BBB permeability was not seen increased in rats with acute hyperglycemia (Hawkins et al., 2007a).

The mechanism of BBB functional impairment in diabetes included the decrease of tight junction (Chehade et al., 2002; Argaw et al., 2009), increase of matrix metalloproteinases (MMPs) in the blood plasma (Hawkins et al., 2007b; Navaratna et al., 2013), oxidative stress (Araki and Nishikawa, 2010; Giacco and Brownlee, 2010), and elevation of inflammatory response (Leal et al., 2010; Wang et al., 2012). However, the pathological mechanism of BBB leakage remained unclear. BBB permeability was measured by means of contrast agents in clinical neuroimaging researches. Most report showed that BBB leakage was detected in patients with mild cognitive impairment or dementia (Mogi and Horiuchi, 2011; Taheri et al., 2011a), which suggested that there was a link between the BBB impairment and the development of cognitive impairment. Although the increased BBB permeability was more frequently detected in patients with cognitive impairment, the relationship between the BBB disruption and diabetes-induced cognitive decline was not conclusive (Mooradian et al., 2005; Aggarwal et al., 2015). In this study, we explore whether the cognitive impairment in the streptozotocin-induced diabetic rat is associated with increased BBB permeability and the change of the inflammatory cytokine.

## Materials and Methods

### Experimental Design

All animal procedures in this study were approved by the Institutional Animal Care and Use Committee of Shanghai Jiao Tong University, Shanghai, China. Adult male Wistar rats (*n* = 105) weighting 200–220 g (around the age of 8 weeks) were used and randomly divided into two groups: diabetic group and normal group. All the rats were conducted cognitive behavior evaluation after 8 weeks. Rats were housed with free access to food and water under a 12 h light-dark cycle (light on at 8:00, light off at 20:00). Rats in the diabetic group were injected with STZ, while rats in the normal group were injected sodium citrate buffer.

**FIGURE 1:**
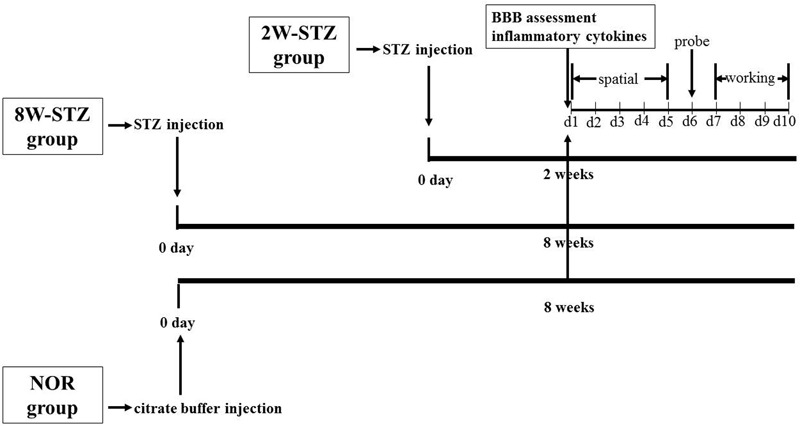
Experimental design. Diabetic models were induced by single injection of streptozotocin on day 0. In the 2-week STZ group, the cognitive assessments were started at 2 weeks after STZ injection. The spatial acquisition trials over the first five consecutive days were followed by probe test on day 6 and working memory test from day 7 to day 10. In the 8-week STZ group, the same cognitive assessments were started at 8 weeks after STZ injection. BBB integrity and inflammatory cytokines were measured at 2 and 8 weeks after STZ injection, respectively. Rats in the normal group were conducted above experiments at 8 weeks after injection of sodium citrate buffer. NOR, normal rats; 2W-STZ, 2-week STZ; 8W-STZ, 8-week STZ. BBB, blood brain barrier; spatial, spatial acquisition trials; probe, probe test; working, working memory test.

To determine the diabetic effect of time course on the BBB permeability and the cognitive function, the diabetic group was further divided into two subgroups according to the time after STZ injection: 2- and 8-week STZ groups. Cognitive function, BBB integrity and inflammatory cytokines were assessed at 2 weeks after STZ injection in the 2-week STZ group. The same experiments were conducted at 8 weeks after STZ injection in the 8-week STZ group (**Figure 1**). We set up a control group of normal rats the same age of STZ groups to eliminate cognitive impairment associated with physiological aging.

### Induction of Diabetes Mellitus in Rats

Diabetes was induced by single intraperitoneal injection of STZ (60 mg/kg body weight, Sigma, St. Louis, MO, United States) according to the previous literature (Hawkins et al., 2007a; Wu and Huan, 2008; Fan et al., 2012; Furman, 2015). STZ was dissolved in 0.1 M sodium citrate buffer (pH 4.5) to a final concentration of 10 mg/ml and used within 20 min of being dissolved. Before STZ administration, rats were fasted (water was available) for 10 h. After STZ injection, rats were allowed to food and water freely. Age-matched non-diabetic normal rats were given 0.1 M sodium citrate buffer intraperitoneally. The serum glucose levels were measured via a blood glucose meter (Bayer HealthCare LLc, Mishawaka, IN, United States) at 7 days, 2, 4, and 8 weeks after STZ injection. If the serum glucose was above 300 mg/dl from 1 to 8 weeks, the rat was considered as diabetics and used for the study.

### MWM Test

The MWM test was performed as described on the nature protocols (Vorhees and Williams, 2006). The experiments included 3 different assessments: spatial acquisition trials, probe tests and spatial working memory. All trials were performed in a quiet room with indirect lighting. The apparatus was a circular tank with 170 cm in diameter as a swimming pool, and contained water at approximately 20–22°C. The swimming pool was virtually divided into four equal quadrants. The edible pigment was used to opaque water that helps to camouflage the submerged platform. The hidden circular platform of 9 cm in diameter located in the quadrant of NE and submerged 1.5 cm below the water surface and remained constant during the entire spatial acquisition trials.

The spatial acquisition trials were conducted over five consecutive days. Each trial had a ceiling time of 2 min with an inter-trial interval of 15 s and four trials per day. At the start, the animal was placed in the designated start position in the maze, facing the sidewalls of the swimming pool. Here we applied start locations of SE, S, NW, W, with a semi-random set as shown in **Table 1**. If the animal failed to reach the platform within 2 min, it was guided to the platform using a guide stick. After the animal reached the escape platform, it was allowed to remain there for 15 s before the beginning of the next trial. The escape latency was defined as the time that the animal spent in climbing onto the escape platform from the start position. The probe test was conducted on the subsequent day 6. In this procedure, the escape platform was removed from the pool and the animals were allowed to swim for 30 s. We recorded the percentage time spent in the target quadrant and the number of times that the animal crossed the zone of the former platform. After the probe test, the spatial working memory was conducted on the next 4 days as previously described (Vorhees et al., 2004; Vales et al., 2006; Vorhees and Williams, 2006; Spritzer et al., 2011). During the test, the platform was moved every day and the animal was given 2 trials on each day (**Table 2**). Trial 1 was the sample trial to learn the new location of the platform, and trial 2 was performed for measurement after a 15 s inter-trial interval through recall and temporary memory. The escape latency in trial 2 was recorded for analysis. The swim path for each trial was monitored by charge coupled device image detectors and quantitatively analyzed by the behavioral tracking software (ANY-maze, Stoelting Co., Wood Dale, IL, United States), search ability was analyzed by the swim path, which were divided into 4 types including straight, tendency, marginal and random by an investigator blinded to experimental groups (Jiang et al., 2004). All the swim paths in spatial acquisition trials were collected for a further analysis of search ability.

**Table 1:** Start positions for the spatial and probe test of Morris water maze.

Day	Trial 1	Trial 2	Trial 3	Trial 4
1	S	W	NW	SE
2	NW	S	SE	W
3	SE	NW	W	S
4	W	SE	S	NW
5	S	NW	W	SE
6(Probe)	SW			

**Table 2:** Platform and start positions in spatial working memory.

Day	Platform	Start position
7	NW	E
8	SE	W
9	NE	S
10	SW	N

### Immunohistochemistry

Rats were sacrificed with a high dose of chloral hydrate (10%) anesthesia. A series of 20 μm coronal sections were cut from the anterior commissure to hippocampus with 200 μm interval for immunostaining. The functional and morphological alterations of BBB were assessed by IgG and tight junction immunostaining. As previously described, VECTASTAIN Universal ABC Kit (Vector Labs, Burlingame, CA, United States) was used to perform IgG staining (Tang et al., 2014). Briefly, brain slices were fixed with 4% paraformaldehyde and blocked with diluted normal serum. Then, slices were incubated with biotinylated secondary antibody and ABC reagent for 30 min, respectively. After that, slices were visually detected immunoreactivity with DAB reagents (Vector Labs, Burlingame, CA, United States) and counterstained with hematoxylin. Five random fields in the brain were photographed in each section. Finally, 25 photographs were collected and analyzed using IPP software (Image Pro Plus 6.0, Media Cybernetics, Bethesda, MD, United States) for mean integrated optical density (IOD) analysis in each rat as previously described (Huang et al., 2013; Tang et al., 2014). For the occludin and claudin-5 staining, brain slices were fixed with methanol for 10 min and then blocked with diluted normal donkey serum (Jackson ImmunoResearch, West Grove, PA, United States). Slides were drained and incubated overnight at 4°C with primary antibodies of vWF (rabbit anti-rat, 1:1000, Abcam, Cambridge, United Kingdom), occludin (mouse anti-rat, 1:200, Life Technologies, Carlsbad, CA, United States), claudin-5 (mouse anti-rat, 1:200, Invitrogen, Carlsbad, CA, United States). After rinsing with PBS, brain sections were further incubated with second antibodies donkey anti-rabbit 488 (1:500, Invitrogen), donkey anti mouse 594 (1:500, Invitrogen) for 1 h at room temperature. Brain sections were photographed using a confocal microscope (Leica, Solms, Germany). At least four vessels per slide and total eight slides from each animal were acquired randomly. We then applied Image J software (National Institutes of Health) to measure the length of the gaps (vWF+/occludin- or vWF+/claudin 5-) and these vessels. Gap length was defined by the ratio of the length of the gap to the whole length of tight junction staining as previously reported (Huang et al., 2013; Tang et al., 2014).

### Real-Time PCR

The brain tissues with 2 mm thick around Willis Circle were isolated and collected to examine the mRNA level of inflammatory cytokines. RNA extraction was conducted by TRIzol reagent (Invitrogen, Carlsbad, CA, United States). After measurement of RNA concentration by spectrophotometer (NanoDrop1000, Thermo, Wilmington, DE, United States), the reverse transcription reaction was performed by a PrimeScript RT reagent kit (Takara, Dalian, China) according to the manufacturer’s instruction. Then cDNA was used to amplify and quantify by SYBR Premix Ex Tag Kit (TaKaRa). The amplification parameters were 95°C for 30 s followed by 40 cycles of 95°C for 5 s and 60°C for 30 s. The measurement was conducted in triplicate. The mRNA level of inflammatory cytokines was normalized to reference gene GAPDH and shown as relative expression of mRNA by 2^-Δct^ method. The primer sequences are listed in **Table 3**.

**Table 3:** Real-time PCR primers.

Gene	Forward primer (5′–3′)	Reverse primer (5′–3′)
TNF-α	TGATCGGTCCCAACAAGGAG	TCCGCTTGGTGGTTTGCTAC
IL-1β	AGTCTGCACAGTTCCCCAAC	TTAGGAAGACACGGGTTCCA
IL-6	GGTTTGCCGAGTAGACCTCA	TACCCCAACTTCCAATGCTC
GAPDH	GATGGTGAAGGTCGGTGTGA	TGAACTTGCCGTGGGTAGAG

### Statistical Analysis

Results were mean ± SD. Data were analyzed by SPSS for both parametric and non-parametric comparisons. A probability value < 0.05 was considered as statistical significance. For analysis of water maze test, results were mean ± SEM. We applied repeated measurement ANOVA for escape latency and logistic regression analysis to analyze search strategies. The immunohistochemistry data were assessed with an unpaired Student’s *t*-test using SPSS 16.0 (SPSS Inc, Armonk, NY, United States).

## Results

### Blood Glucose After STZ Injection

Compared to the normal group, the blood glucose of the STZ groups increased significantly on 7th day and maintained at a high level during the 8 weeks after STZ injection (**Table 4**, *p* < 0.01).

**Table 4:** Glucose measurements in normal and diabetic rats.

	Blood glucose (mg/dl)				
	0 day	7 days after	2 weeks after	4 weeks after	8 weeks after
		injection	injection	injection	injection
normal group	95 ± 22	93 ± 24	88 ± 22	95 ± 25	97 ± 21
2-week STZ group	93 ± 18	458 ± 38*	461 ± 27*	/	/
8-week STZ group	92 ± 24	451 ± 32*	463 ± 45*	470 ± 46*	473 ± 25*

*Rats in normal group were given 0.1 M sodium citrate buffer intraperitoneally. Values are mean ± SD, *n* = 32–40 per group. ^∗^*p* < 0.01.*

### Cognitive Performance of Diabetic Rats Was Worse in MWM Test

**FIGURE 2:**
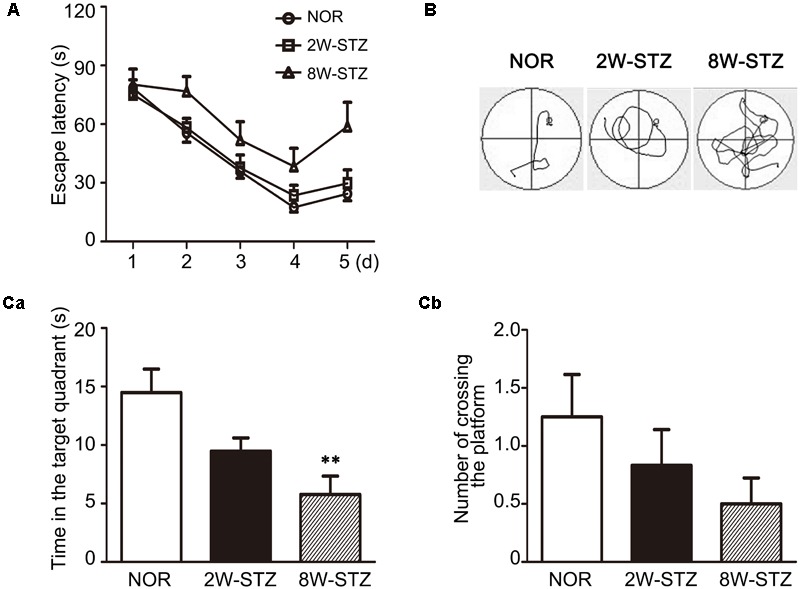
Cognitive performance of diabetic rats was worse in acquisition trial and probe test of Morris water maze test. **(A)** Line graphs showed the change in escape latency from day 1 to day 5 among 2W-STZ, 8W-STZ and normal groups. The sequence of start position was shifted each day and the hidden platform was fixed in the quadrant NE. *p* = 0.003, 8W STZ vs. normal rats. *p* = 0.028, 2W-STZ vs. 8W-STZ. **(B)** The track plot showed the trace of rats from the start position to the platform (circle) in the tank during the spatial acquisition trials. **(C)** The platform was removed in the probe test. Bar graph showed the time spent in the target platform quadrant **(Ca)** and the number of crossing the original platform area **(Cb)**. The time spent in the target quadrant of 8-week STZ rats significantly decreased **(Ca)** compared to the normal rats. There was no difference in the number of crossing the platform among groups **(Cb)**. Data are mean ± SEM, *n* = 6–8 per group, ^∗∗^*p* < 0.01, 8W-STZ vs. normal rats. NOR, normal rats; 2W-STZ, 2-week STZ; 8W-STZ, 8-week STZ.

**FIGURE 3:**
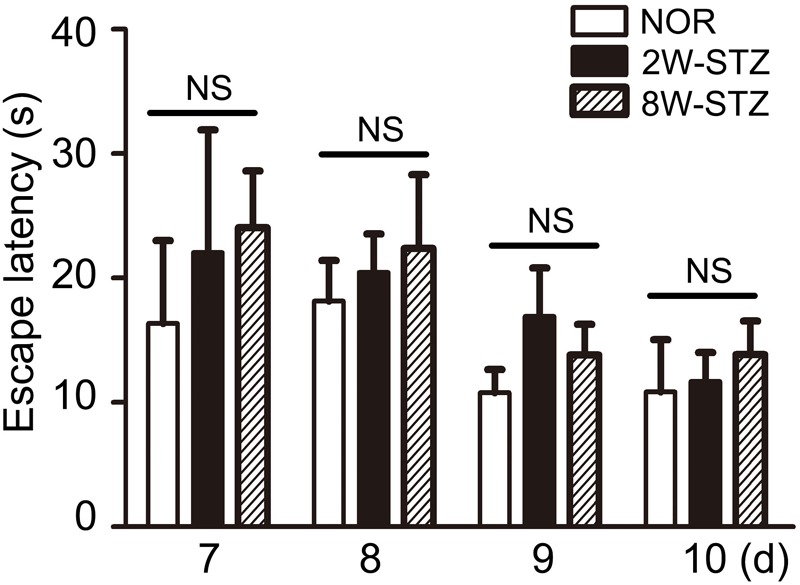
Diabetic rats’ performance in working memory was not worse. Working memory test was conducted from day 7 to day 10 in the cognitive evaluation. Working memory was to conduct a consecutive four-day experiment and two trials a day. We set trial 1 as a sample trial, bar graph showed no statistically significant difference in trial 2 among the diabetic and normal rats. Data are mean ± SEM, *n* = 6–8 per group. NOR, normal rats; 2W-STZ, 2-week STZ; 8W-STZ, 8-week STZ. NS, no significance.

We used the Morris water maze test to evaluate cognitive function among the different groups. Spatial acquisition trials were conducted on the first 5 days of cognitive evaluation. The escape latency of 8-week STZ group was increased compared to the normal rats (*p* < 0.05), and there was no significant difference between 2-week STZ group and the normal rats (**Figures 2A,B**). Furthermore, the probe test on the 6th day of cognitive evaluation showed that the time spent in the target quadrant of 8-week STZ rats was significantly decreased compared to the normal rats (*p* < 0.05), accompanied by the reduction in the number of former platform crossing. While no difference was detected between the 2-week STZ group and the normal rats. These findings suggested that the spatial memory was impaired in the 8-week STZ rats and spared in the 2-week STZ rat (**Figure 2C**). However, there were no significant differences in the performance of the spatial working memory test among groups (**Figure 3**).

### Diabetic Rats Showed Ineffective Search Ability

**FIGURE 4:**
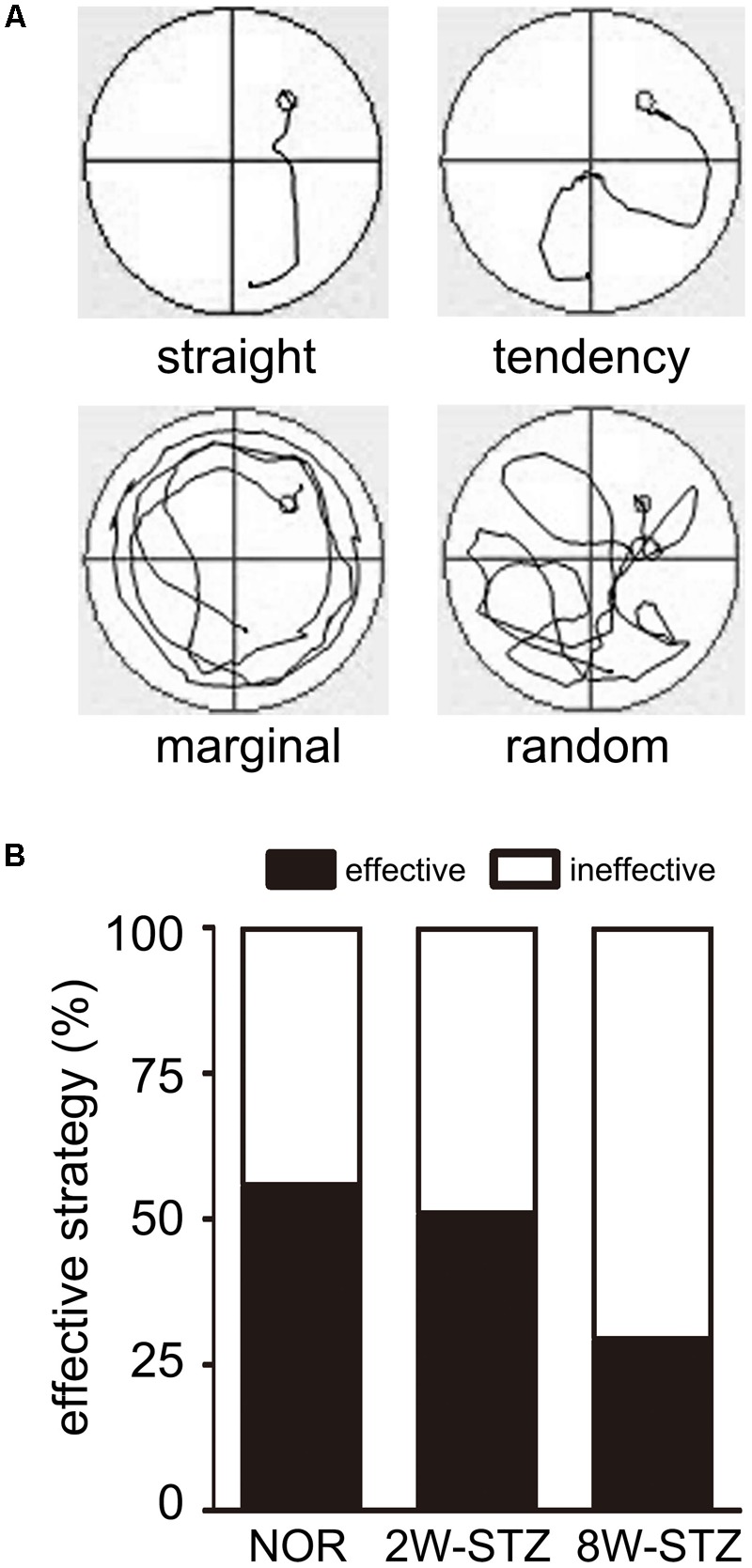
The normal rats applied more effective ability compared to diabetic rats. **(A)** The swim paths for each trial were monitored by charge coupled device image detectors and divided into 4 types including straight, tendency, marginal and random by an investigator blinded to experimental groups. Different swim paths showed different search abilities in the experiment. Straight and tendency are considered as the effective ability, while marginal and random are considered as the ineffective ability. **(B)** Bar graphs showed the 8W-STZ rats preferred an ineffective ability compared with that of the normal rats and the 2W-STZ rats (*p* < 0.01). *n* = 6–8 per group. NOR, normal rats; 2W-STZ, 2-week STZ; 8W-STZ, 8-week STZ.

We then examined search abilities in spatial acquisition trials. Straight and tendency swim paths were defined as an effective ability, while marginal and random were defined as the ineffective ability. We found that the 8-week STZ rats preferred ineffective ability, including marginal and random. Meanwhile the normal rats and the 2-week STZ rats were more likely to use an effective ability (*p* < 0.01, **Figure 4**). After logistic regression analysis, 8 weeks after STZ injection was independent predictors of ineffective ability (*p* < 0.01).

### Diabetes Increased IgG Leakage

**FIGURE 5:**
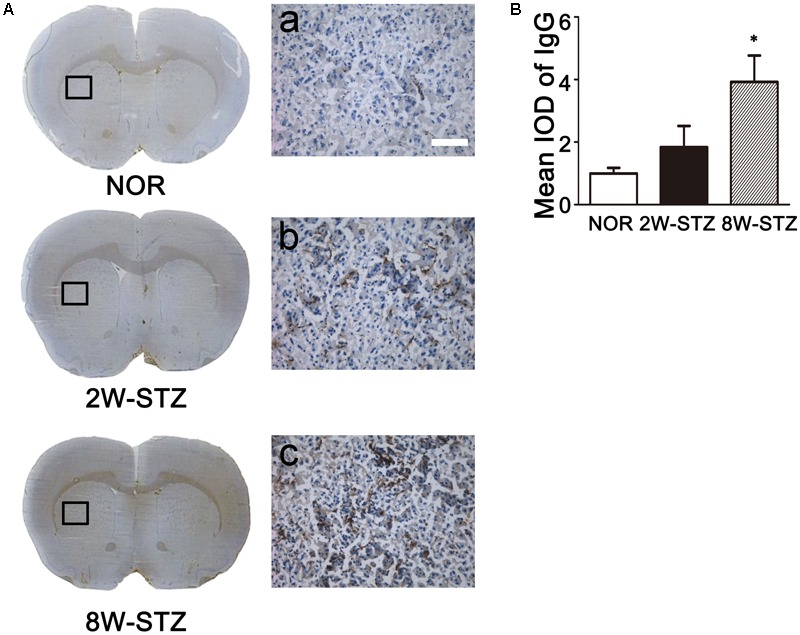
Leakage of IgG in diabetic and normal rats. **(A)** Photomicrographs showed IgG staining in the diabetic and normal rats. Brown signal was the IgG positive protein leaked from the vessel. Boxes in images of NOR, 2W-STZ, and 8W-STZ are magnified to a. b and c, respectively, bar = 100 μm. **(B)** Bar graph showed the quantification of IgG protein in the different groups. Data are mean ± SD, *n* = 6–8 per group. ^∗^*p* < 0.05, 8W-STZ vs. NOR rats. NOR, normal rats; 2W-STZ, 2-week STZ; 8W-STZ = 8-week STZ; IOD, integrated optical density.

BBB permeability was evaluated by IgG leakage. IgG leakage was detected at 2 and 8 weeks after STZ injection. We found that IgG extravasation was significantly increased in the 8-week STZ rats compared to the normal rats and 2-week STZ rats (**Figure 5**), which indicated that BBB permeability was increased in the 8-week STZ rats.

### The Gap in the Tight Junction Was Increased in Diabetic Rats

**FIGURE 6:**
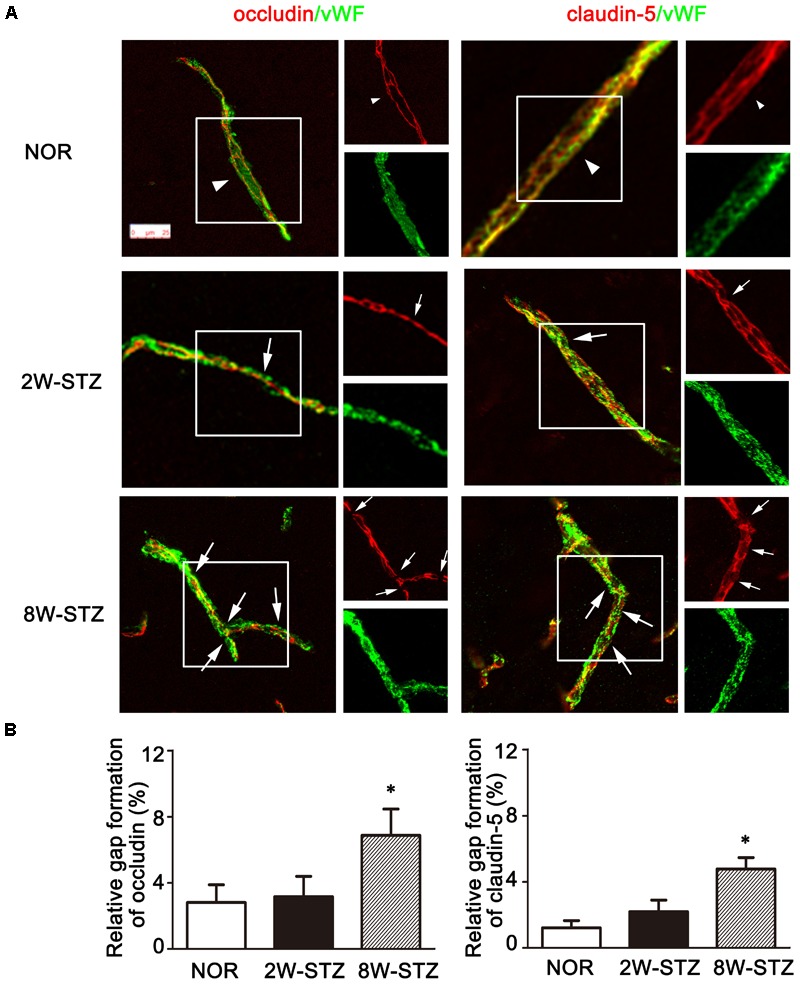
Tight junction of Occludin and claudin-5 in the diabetic and normal rats. **(A)** Representative image of tight junction protein occludin and claudin-5 (red) showed continuous and linear expression along the vessel labeled with endothelial marker vWF (green) in the normal rats (arrowheads). Compared to the normal rats, the occludin and claudin-5 protein showed a discontinuous distribution in 8-week STZ rats (arrows). The box section of each large image was shown in the small figure on the right side. Scale bar = 25 μm. **(B)** Bar graph showed the ration of the gap length to the vessel length among different groups. Data are mean ± SD, *n* = 6–8 per group. ^∗^*p* < 0.05, 8W-STZ vs. normal rats. NOR, normal rats; 2W-STZ, 2-week STZ; 8W-STZ, 8-week STZ.

To further investigate the mechanism of BBB leakage, we performed vWF/occludin and vWF/claudin-5 double staining. The results showed that the expression of occludin and claudin-5 decreased and the gap enlarged and discontinued in the 8-week STZ rats compared to the normal rats (*p* < 0.05, **Figure 6** and Supplementary Figure S1). However, these morphological changes were not detected in the 2-week STZ rats.

### Diabetes Increased the IL-6 mRNA Level in the Brain

**FIGURE 7:**
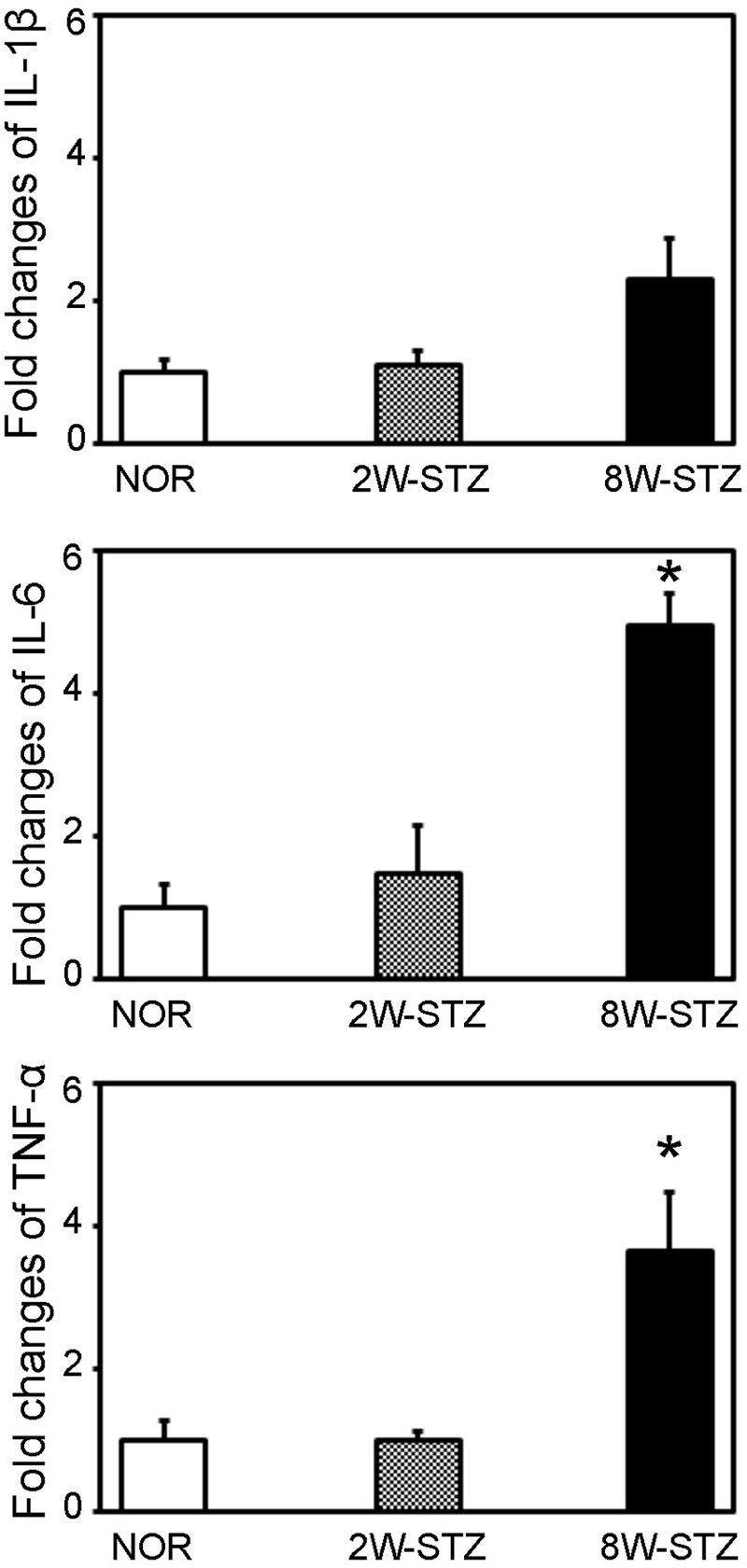
The mRNA level of inflammatory cytokines in the diabetic and normal rats. Bar graph showed the quantification of TNF-α, IL-1β and IL-6 mRNA level in the normal, 2- and 8-week STZ rats. Data were normalized to the internal control. Data are mean ± SD, *n* = 4 per group. ^∗^*p* < 0.05, 8W-STZ vs. normal rats. NOR, normal rats; 2W-STZ, 2-week STZ; 8W-STZ, 8-week STZ.

To determine whether the inflammatory cytokines was involved in the cognitive impairment of the diabetic rats, we examined TNF-α, IL-1β and IL-6 mRNA expression in the brain. We found that TNF-α and IL-6 mRNA were increased in the brain of 8-week STZ rats compared to the normal rats (*p* < 0.05, **Figure 7**). However, there is no difference in the mRNA level of inflammatory cytokines between the normal and 2-week STZ rats.

## Discussion

Our results demonstrated that the increase of BBB permeability and the decrease of cognitive function was consistent with the progression of diabetes, which implicated that there was a relationship between BBB leakage and cognitive impairment in diabetic animal. BBB impairment might play a role in the mechanism of cognitive dysfunction in diabetes.

Diabetes mellitus has been demonstrated to increase the risk of cognitive impairment, including vascular and neurodegenerative forms (Velayudhan et al., 2010; Singh-Manoux and Schmidt, 2015). The cognitive dysfunction in patients with type 1 diabetes was characterized by reduced performance on flexibility and mental speed (Brands et al., 2005; McCrimmon et al., 2012). The duration of the disease is the key factor for the incidence of dementia (Ryan et al., 2003).

Morris water maze test is commonly used to evaluate the cognitive function in rodents. Various test protocols are available for the detecting cognitive function. In our study, fixed hidden platform, fixed start location and 15 s-inter-trial interval were applied in working memory test, which only assessed the temporary or working memory. In the spatial acquisition and probe test, the start position was changeable, and the probe trial was conducted 24 h after the last acquisition trial. Animals used the distal cues, like plastic board in different colors and shapes around the circular tank, to navigate an optimal path from the start location to the submerged platform. In this process, working memory, task flexibility, reasoning, and planning and execution were all assessed. We found a worse performance in the spatial acquisition trials and probe test in the 8-week STZ rats compared to the normal rats. Moreover, analysis of search ability showed that fewer 8-week STZ rats preferred to apply straight or tendency ability compared to the normal or the 2-week STZ rats. These results indicated that normal rats could focus on the task and apply distal cues to navigate their way to escape platform after learning. Eight-week STZ rats, however, tended to be more thigmotaxic and purposeless, and use distal cues less to accomplish the task. In working memory test, however, all the rats exhibited a normal spatial working memory, which suggested that immediate memory remained intact in the diabetic rats. Taken together, the cognitive impairment was gradually obvious with the progress of diabetes in the diabetic rats. Given the different results among spatial acquisition trials, probe test and working memory, we believed the main feature of cognitive dysfunction in the 8-week STZ rats was the executive dysfunction, rather than the temporary memory deficits. Working memory decline was evident in patients with Alzheimer’s disease (Hort et al., 2010; Sorbi et al., 2012), and working memory was also damaged using the evaluation of MWM tests in mouse model of Alzheimer’s disease (Janus, 2004). Meta-analysis showed the executive dysfunctions featured in patients with diabetes and no difference of working memory between the diabetics and non-diabetics (Sadanand et al., 2016).

Hippocampus is an important structure which is in charge of memory and learning. But memory impairment is not only associated with hippocampus. BBB disruption has been proved to be involved in the pathogenic mechanism of many neurological diseases such as stroke, multiple sclerosis, epilepsy and dementia (Abbott et al., 2010; Wang et al., 2016; Yang et al., 2017). Increasing evidence shows that BBB damage may participate in the pathophysiology of cognitive decline and dementia. BBB permeability can be measured by means of contrast agent in clinical neuroimaging researches. And most of them reported that BBB leakage was detected in patients with mild cognitive impairment or dementia (Hanyu et al., 2002; Starr et al., 2009; Taheri et al., 2011a,b), suggesting a link between BBB damage and cognitive impairment. Recently, studies have paid much attention on the cerebral microangiopathy caused by diabetes, and the function of BBB has become the specific target for the evaluation of endothelium function. Due to different animal models and specific vascular space markers, changes of BBB permeability in experimental diabetic animals were inconsistent (Acharya et al., 2013; Shao and Bayraktutan, 2013). There are various tracers to detect BBB permeability at different time points after STZ injection (Huber et al., 2006; Hawkins et al., 2007a). In our study, we performed IgG as a tracer to assess the BBB permeability as previously described (Huang et al., 2013; Tang et al., 2014), and detected that BBB permeability increased as the duration of hyperglycemia. The BBB damage gradually accelerated in the meanwhile the cognitive dysfunction in the 8-week STZ rats aggravated compared to the 2-week STZ rats, which was consistent with our hypothesis that the severity of cognitive function might be correlated with BBB permeability.

The tight junction between the cerebral endothelial cells are the critical structure for maintaining BBB function. In our study, we demonstrated that the gap in the tight junction of the 8-week STZ rats was significantly larger than that in the normal rats, which suggested that tight junction protein degradation was responsible for the disruption of BBB function. We also found that the significant higher level of TNF-α and IL-6 in the brain of 8-week STZ rats compared to the normal or 2-week STZ rats, which suggested that the inflammation participated in the process of the disease. Other studies also demonstrated inflammation were involved in tight junction decomposition and BBB disruption. The inflammation after stroke affected the expression of tight junction proteins and increased BBB permeability, which led to the exacerbation of the disease (Fernandez-Lopez et al., 2012). The inflammatory responses provoked distortion and decrease of tight junction protein and triggered visible extravasation of IgG in C57/Bl6 mice of experimental autoimmune encephalomyelitis (Shrestha et al., 2014). The elevation of MMP-2 induced by IL-6 signaling was reported in the patients with BBB disruption of neuromyelitis optica (Uchida et al., 2017). The IL-6 was also reported associated with cognitive behavior disorder in clinical study. Patients with neuropsychiatric systemic lupus erythematosus was found significant elevation of IL-6 concentrations in the CSF (Asano et al., 2017). A recently study found the production of IL-6 activated by TNF-α elevated the permeability of brain endothelial cells *in vitro* (Rochfort et al., 2016). Taken together, we considered that diabetes might increase the level of TNF-α and IL-6 and trigger the degradation of tight junction and the disruption of BBB in the brain, which eventually caused cognitive impairment. The future studies should assess whether the BBB disruption and cognitive impairment are alleviated by blocking the TNF-α or IL-6 signaling.

## Author Contributions

JG conceived of the study, collected data, performed the statistical analysis, and drafted the manuscript. LW participated in the design of the study, carried out immunohistochemistry and neurobehavioral tests. LZ, CQ, and YS participated in the assessment of cognitive function. YC carried out mRNA assay. SC participated in the design of the experiments. YW and ZZ helped to design the experiment, interpret the data, and draft the manuscript. G-YY inspected all experiments for rationality and scientificity, provided financial support, supervised and revised the manuscript. All authors read and approved the final manuscript.

## Conflict of Interest Statement

The authors declare that the research was conducted in the absence of any commercial or financial relationships that could be construed as a potential conflict of interest.
