# Supplementary material for: Blood-Brain Barrier Disruption Induced Cognitive Impairment Is Associated With Increase of Inflammatory Cytokine
Source: Front Aging Neurosci. 2018 May 7;10:129. doi: 10.3389/fnagi.2018.00129 (PMC5949351; doi:10.3389/fnagi.2018.00129)
Supplement: Supplementary file 1 [file Data_Sheet_1.PDF]

Blood-brain barrier disruption induced cognitive impairment is associated with increase of inflammatory cytokine

Figure S1.

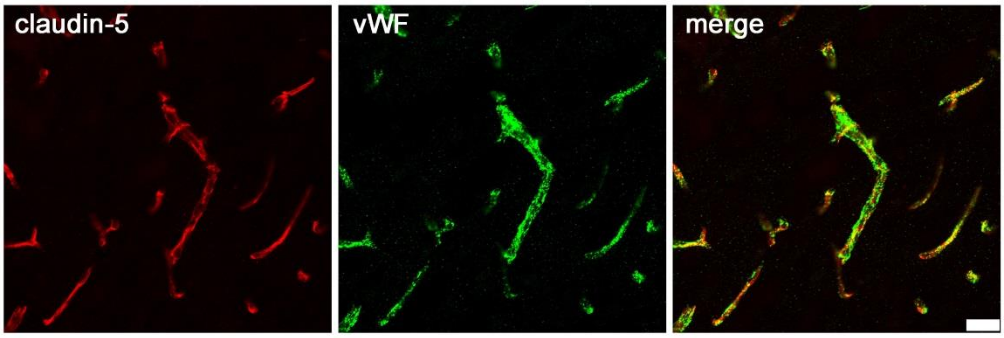

Figure S1. The original picture of blood vessel staining in brain. Scale bar = 25μm.
